# Mild Microfluidic Approaches to Oxide Nanoparticles Synthesis

**DOI:** 10.1002/chem.202103132

**Published:** 2021-12-16

**Authors:** Paolo Zardi, Tommaso Carofiglio, Michele Maggini

**Affiliations:** ^1^ Department of Chemical Sciences University of Padova Via Francesco Marzolo 1 35131 Padova Italy

**Keywords:** continuous flow, low temperature synthesis, microfluidic, nanoparticles, oxide

## Abstract

Oxide nanoparticles (oxide NPs) are advanced materials with a wide variety of applications in different fields. The use of continuous flow methods is particularly appealing for their synthesis due to the high control achieved over the reaction conditions and the easy process scalability. The present review focuses on the preparation of oxide NPs using microfluidic setups at low temperature (≤80 °C), since the employment of mild reaction conditions is crucial for developing sustainable and cost‐effective processes. A particular emphasis will be put on the improvement over the final product features (e. g., size, shape, and size distribution) given by flow methods with respect to conventional batch procedures. The main issues that arise by treating NPs suspensions in microfluidic systems are product deposition or channel clogging; mitigation strategies to overcome these drawbacks will also be presented and discussed.

## Graphical abstract

**This review reports** the preparation of oxide nanoparticles in microreactors under continuous flow conditions. The use of suitable methodologies allows to manage the size and shape of the nanoparticles, through the fast mixing of the reagents and the precise control of the reaction conditions that can be achieved within flow devices, if compared to batch syntheses.

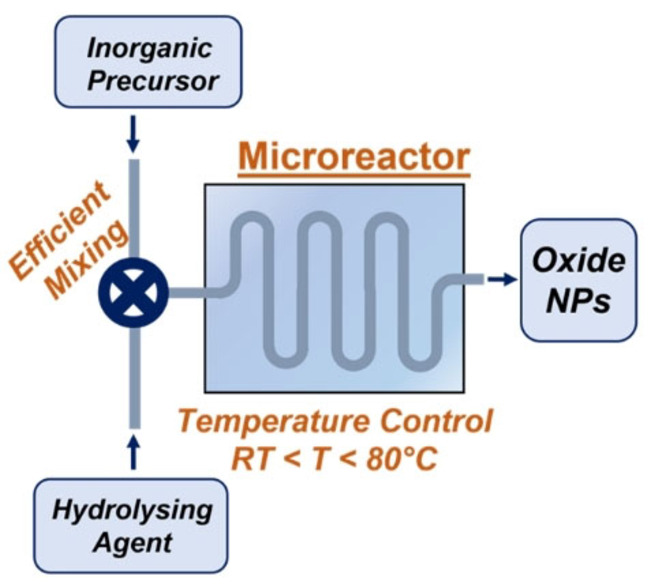

## 1 Introduction

The production of inorganic nanomaterials with tailored functional properties represents a breakthrough in materials science with the promise of significantly changing, in the near future, strategical fields such as energy conservation, textiles, optoelectronics, healthcare, catalysis, cosmetics, semiconductors, bioimaging, and chemical sensing, to name a few.^[1]^

Functional properties of molecular‐based materials can be changed on demand by wisely exploiting the stereo‐electronic effects of the substituents. Nanomaterials, on the other hand, exhibit fascinating electronic, optical, thermal, magnetic, and chemical properties, that are significantly different from those of their bulk counterparts, resulting from the possibility to produce nanoparticles (NPs) with predetermined morphology and size distribution. This control can be accomplished by optimizing the experimental parameters influencing NPs nucleation and growth.^[2]^

Traditional solution‐based syntheses for the *“chemie douce”* preparation of gram‐scale amounts of NPs are frequently carried out into 10–100 mL batch reactors. To reproducibly obtain high‐quality NPs with a narrow size distribution, experimental parameters such as reagent concentration, temperature, mixing time, and reaction duration must be optimized and rigorously standardized through extensive investigation.^[3]^ In many cases, the synthetic protocols turn out to be energy and time‐consuming often entailing hazardous and environmental adverse chemicals. Consequently, many of the reported procedures for the production of valuable NPs are not straightforwardly amenable to cost‐effective, simple to implement processes to prepare materials of consistent quality. Moreover, scaling up NPs production is inherently problematic. Indeed, increasing the reactor volume and/or reagents amount to manufacture NPs in a suitable scale to supply the commercial demand inevitably triggers temperature and concentration gradients across the reactor. This induces significant polydispersity and batch‐to‐batch variability as a result of the high distribution of nucleation and growth rates.

Continuous flow processing is a consolidated technique to increase organic synthesis efficiency^[4]^ which is now recognized as a valuable tool also for the preparation of inorganic nanomaterials.^[5]^ The principal benefit associated to chemical reactions carried out in flow within narrow‐bore tubing is ascribed to the large surface‐to‐volume ratio that allows a precise and reproducible control of heat and mass transfer events. More importantly for large‐scale production, syntheses can be scaled up by running a flow reaction for an extended period of time or by using multiple reactors working in parallel. Both methods do not require a re‐optimization of the reaction conditions, thus speeding up the process to meet a required output. Further advantages are a safer handling of hazardous chemicals and intermediates, the straightforward integration of analytical tools for real‐time reaction monitoring, the possibility of in‐line purification and reagent introduction at specific check points, in the case of a multistep synthesis. Also, the miniaturization of reactors reduces the use of starting material during the optimization phase, when the experimental parameters need to be investigated systematically over a wide range of values.

A common opinion, which has probably discouraged the application of continuous flow reactors for NPs synthesis, is their supposed inadequacy to handle insoluble materials that may cause channel clogging or fouling. We hope that this review will help the reader not only to overcome this preconception but also to understand the opportunities offered by continuous flow methodologies for the synthesis of oxide NPs. These methods will be summarized defining the underlying principles and motivations for their use. Then, a survey for the production of the most common inorganic oxide NPs (i. e., iron, zinc, silicon, titanium, zirconium, cerium, copper, and magnesium oxides) will be given, with special attention to those syntheses carried out at moderate (i. e. 80 °C) temperatures that are likely the most appealing from the point of view of sustainability and industrial applications.

## 2 Flow Methodologies

The advantages of a continuous flow approach for the controlled synthesis of NPs can be qualitatively rationalized considering the classical nucleation theory (LaMer model^[6]^ and later advancements) which predicts that the dimension of the NPs is determined mostly by the ratio of the supply rate of the precursors over the growth rate. A first improvement given by a continuous flow processing, if compared to batch synthesis, is a better control over the spatial concentration of NPs precursors. A fine tuning of the reactants flow rates, coupled with an efficient mixing that in a flow reactor minimizes concentration gradients, affords a homogeneous chemical environment at a proper concentration for the nucleation to take place. The timing of the subsequent NPs growth process can be also carefully controlled by a proper choice of the residence time in the reactor. Therefore, the superior degree of control given by flow reactors can be considered, for NPs synthesis, as an important factor to tune the output of the reaction in terms of particles size and size distribution.

The continuous flow methods described below are the most frequently used to prepare oxide NPs that, generally, form by a rapid mixing of stream **A**, containing the inorganic precursor (a metal salt or an alkoxide species) with stream **B**, containing a hydrolyzing agent (usually a basic aqueous solution). The nucleation and evolution of the NPs in flow is controlled by two main strategies: a) the subdivision of the reacting mixture stream in well‐confined spaces and b) the generation of recirculation motions in the stream. In this way, NPs form in a reproducible manner. As far as the microreactors materials are concerned, polydimethylsiloxane (PDMS) chips made by soft lithography are suitable for reactions in aqueous or alcoholic media at mild temperatures. In addition, microreactor chips made with other polymeric materials (e. g., polyacrilates, polyketones) and coil reactors built from tubing of fluorinated polymers (e. g. PTFE) or stainless‐steel have been also used for the production of NPs in more aggressive media.

Nevertheless, NPs production under continuous flow conditions is associated to the issue of clogging and fouling of the reactor channels, the latter phenomenon being the main problem for the nanostructured materials considered herein. The general strategies for fouling mitigation inside microstructured devices have been reviewed recently.^[7]^ Concerning the NPs oxides preparation, the main anti‐fouling methods rely on avoiding contact of the NPs with the reactor walls (e. g., by performing the reactions in a monodispersed droplet flow or focusing the particulate stream along the channel center).

### 2.1 Flow Injection Synthesis (FIS)

**Figure 1:**
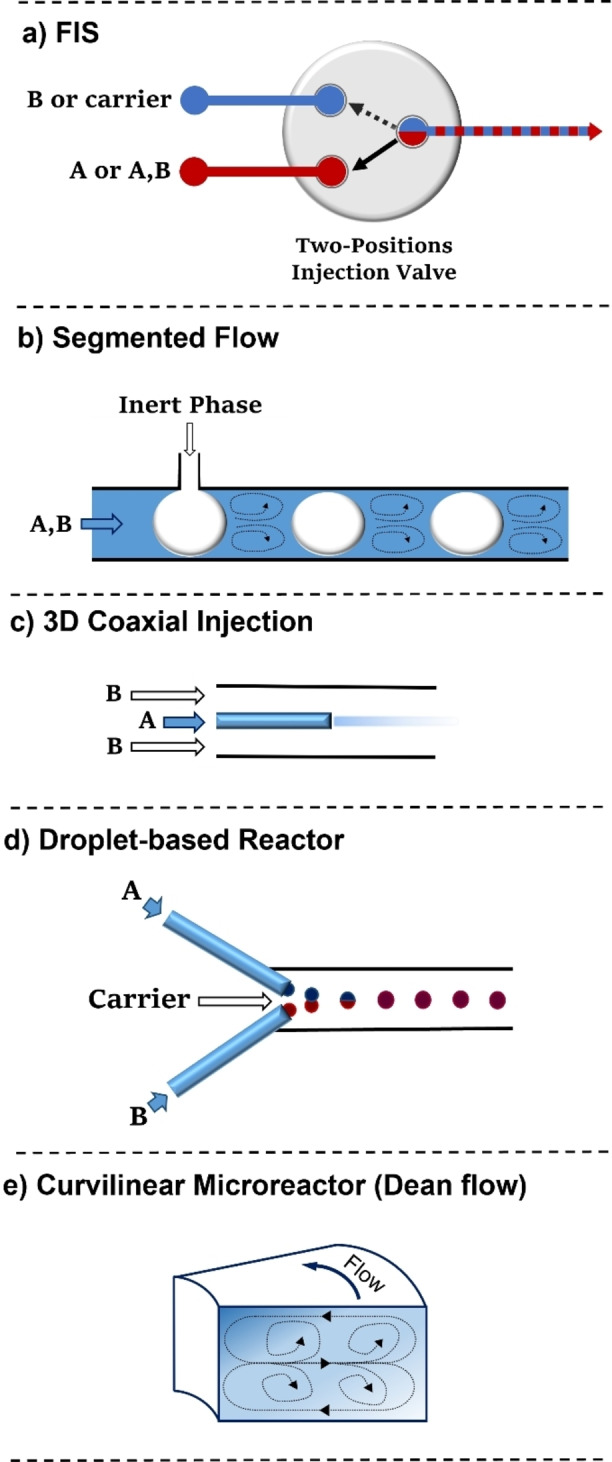
Schematic representation of different flow reactor sections for the preparation of oxide NPs.

This is an early strategy derived from the analogue analytical technique (Flow injection Analysis).^[8]^ It consists in injecting precise volumes of a reagent solution (**A** or **A/B**) into a carrier stream, which may be an inert or another reagent solution (**B**) (Figure 1a). In this way, only a determined amount of **A** is allowed to react at a time. A distinctive feature of the system is the injection valve that switches between the two streams, controlling the volume and frequency of reagent injection into the carrier stream. Despite the conceptual simplicity of this technique, it requires an automated control on the injection valve to achieve reproducible results.

### 2.2 Segmented Flow

This methodology entails the discrete division of a premixed reagent flow (**A** and **B**) in small segments through the intercalation of an immiscible fluid (liquid or gaseous) (Figure 1b). The NPs formation is thus confined in a small space where an efficient mixing is provided by an internal recirculation in each slug, which can be considered as single microreactors. The use of a segmented flow prevents the axial dispersion phenomenon,^[9]^ namely, the flow rate variation from the tube center to the channel wall. This gradient occurs in laminar flow regimes that should be avoided to have a narrow residence time distribution and therefore an enhanced size uniformity of the NPs. The pre‐mixing the A and B streams before the inert fluid intercalation may limit the application of this technique to the synthesis of oxide NPs not involving a highly fast hydrolysis process (e. g. silica NPs, see Section 4.1).

### 2.3 3D Coaxial Injection

A stream of solution **A** is injected through a coaxial thin tube inside the channel where solution **B** is flowing (Figure 1c). By modulating the rate ratio between the inner and outer flow (Q_OUT_/Q_IN_) it is possible to focus or defocus the inner stream into the channel center. The gradual diffusion of **A** into **B** provides a controlled mixing in the area between the two streams. The inorganic precursor solution flows in the coaxial tube to avoid contact of the NPs with the channel wall, allowing to mitigate microreactor fouling. This methodology is particularly suitable for NPs synthesis involving fast nucleation processes since the mixing time can be highly reduced through the flow rates modulation. Moreover, it allows to confine the NPs formation area without the use of an immiscible fluid and additives (e. g. surfactants). This strategy was originally reported for iron NPs synthesis,^[10]^ and later used for TiO_2_ ^[11]^ and ZrO_2_ ^[12]^ NPs preparation, although for this latter application **A** and **B** were immiscible solutions to localize the nucleation only at the interphase between Q_OUT_ and Q_IN_.

### 2.4 Droplet‐based Reactors

In these reactors, the reagent mixture is distributed within monodispersed small droplets inside a carrier flow of an immiscible solvent (Figure 1d). Each droplet acts as a single microreactor containing a defined quantity of reagents. This methodology benefits of a fast mixing given by internal recirculation inside the droplet and an accurate control over the particles size due to the limited amount of reagent available. Droplets containing both reagents (**A** and **B)** may be directly formed or **A** and **B** can be confined as separated droplets to be paired and united along the reactor channels. A precise and reliable system for droplets generation is required to guarantee NPs size and shape uniformity. A small amount of surfactant is generally needed as droplet stabilizer, although this may lead to contamination of the final NPs. Aqueous droplets are usually flowed into a hydrocarbon or perfluorinated solvent phase; this method avoids a direct contact of **A** and **B** with the channel walls, minimizing the risk of NPs deposition. In addition, the small droplet dimensions further increase the efficiency of mass/heat transfer processes given by the high surface‐to‐volume ratio of flow reactors. This technique is one of the most frequently employed for the synthesis of different oxide NPs (e. g., iron, silica, zinc, titania) because of its control over the particles size and dispersion and the efficient fouling prevention.

### 2.5 Curvilinear Microreactors

The use of microreactors with a curvilinear channel geometry can improve further the mixing time of solutions **A** and **B** thanks to the secondary flows generated by the so‐called Dean instability.^[13]^ This phenomenon occurs at high flow rates and consists in the formation of two counter‐rotating transverse vortices originated by the centrifugal forces inside the flow in a curved channel (Figure 1e). Microreactors with a spiral channel geometry therefore benefit from highly rapid mixing, which, in particular, enhances the control over NPs nucleation. Furthermore, the fluid circulation, which is operative in a curvilinear reactor, prevents the axial dispersion phenomenon mentioned above. The use of a curvilinear reactor can be a simple approach to improve mixing and reproducibility without the need of using more complex setups. However, it is usually combined with other techniques (e. g. droplet reactors) to mitigate fouling of the channels.

## 3 Iron Oxide Nanoparticles

Iron oxide NPs exist in different species depending on the metal oxidation state and crystalline morphology. Among them, the most common iron oxide NPs are crystalline polymorphs of ferric oxide, hematite (α‐Fe_2_O_3_) or maghemite (γ‐Fe_2_O_3_), and magnetite (Fe_3_O_4_), the latter containing both Fe(III) and Fe(II) in a 2 : 1 ratio. Thanks to their superparamagnetic and biocompatible nature, magnetite and maghemite NPs are used for drug delivery and as therapeutic/diagnostic agents.^[14]^ Their solution‐based, batch, synthesis usually involves a co‐precipitation process,^[15]^ where an acidic aqueous solution, containing, for instance, Fe(II)/Fe(III) halides, is mixed with a base (e. g. NaOH or ammonia) leading to immediate precipitation of hydrophilic NPs at room temperature or under moderate heating. The synthesis of pure magnetite NPs requires air exclusion to prevent the oxidation of Fe(II) by oxygen. The batch method is fast and low‐cost but suffers from scarce reproducibility and control over NPs size distribution with respect to high‐temperature methods, such as thermal decomposition of organometallic precursors^[16]^ or hydrothermal synthesis.^[17]^

The use of microreactor technology can be an efficient remedy to the above‐mentioned drawbacks, as pictured by the examples reported in Table 1. By confining the precipitation reaction in a small volume, the polydispersity of the NPs can be efficiently limited, although the channels clogging could be an issue since the fast co‐precipitation process is difficult to control and may cause product deposition over the channel walls.

**Table 1:** Iron NPs syntheses in continuous flow.

Oxide	Reactants	Methodology	Year	T	Shape	Size^[a]^ [nm]	Size distribution [σ_d_/d]	Ref.
Fe_3_O_4_	FeCl_2_/FeCl_3_+NaOH	FIS	2006	80 °C	spherical	3.3	0.23	[18]
γ‐Fe_2_O_3_	FeCl_2_/FeCl_3_+TMAOH	3D‐flow injection	2008	RT	spherical	7	0.2	[10]
α‐FeO(OH)	FeCl_3_+TMAOH	3D‐flow injection+coil reactor	2009	RT/60 °C	plates	l=30 w=7	0.57 0.57	[19]
Fe_3_O_4_	FeCl_2_/FeCl_3_+NH_4_OH	3D‐flow injection	2018	RT	spherical	24.6	0.29	[20]
Fe_3_O_4_	FeCl_2_/FeCl_3_+NaOH	3D‐flow injection	2020	RT	–	8.6^[b]^	0.41	[22]
Fe_3_O_4_	FeCl_2_/FeCl_3_+NH_4_OH	droplet‐based reactor	2008	RT	spherical	4	0.25	[23]
Fe_3_O_4_/γ‐Fe_2_O_3_ (w/dextrane coating)	FeCl_2_/FeCl_3_+NH_4_OH	droplet‐based reactor	2012	RT	spherical	3.6	0.22	[24]
Fe_3_O_4_	FeCl_2_/FeCl_3_+NH_4_OH	droplet‐based reactor	2018	70 °C		10.5	0.17	[25]
Fe_3_O_4_	FeCl_2_/FeCl_3_+NaOH	droplet‐based spiral reactor	2019	RT	–	7	–	[26]
Iron oxide	FeCl_2_/FeCl_3_+NaOH	actively mixed microreactor chip	2009	RT	–	5.24	0.16	[28]
Fe_3_O_4_	FeCl_2_/FeCl_3_+NH_3_(g)	continuous flow SDP	2008		spherical	10^[c]^	–	[27]
Fe_3_O_4_/γ‐Fe_2_O_3_	FeCl_2_/FeCl_3_+NaOH	PTFE coiil reactors	2020	60 °C		6‐8	0.13–0.19	[29]

[a] Calculated by TEM/SEM analysis. [b] At a 1 : 1 Fe3+/Fe2+ ratio. [c] At the optimized conditions (25 °C, grooved disc, 500 rpm) in the presence of alginic acid.

### 3.1 Flow injection synthesis

The first continuous flow synthesis of iron oxide NPs by co‐precipitation was reported in 2006^[18]^ within a flow injection (FI) system where a solution of a FeCl_2_/FeCl_3_ mixture was injected into a continuous stream of aqueous NaOH. The reaction was carried out at 80 °C under inert atmosphere and a residence time of 10 s. Variation of reagent concentration or flow rates revealed an impact on NPs morphology. In particular, a base concentration up to 10 M gave NPs with an average size that is smaller than that of NPs prepared in batch (3.3 nm vs. 4.3 nm) and a better size dispersion. The product was identified as magnetite by thermal analysis, which showed an exothermic peak for the oxidation of Fe_3_O_4_ to γ‐Fe_2_O_3_ at 450 °C.

### 3.2 3D‐Coaxial injection

**Figure 2:**
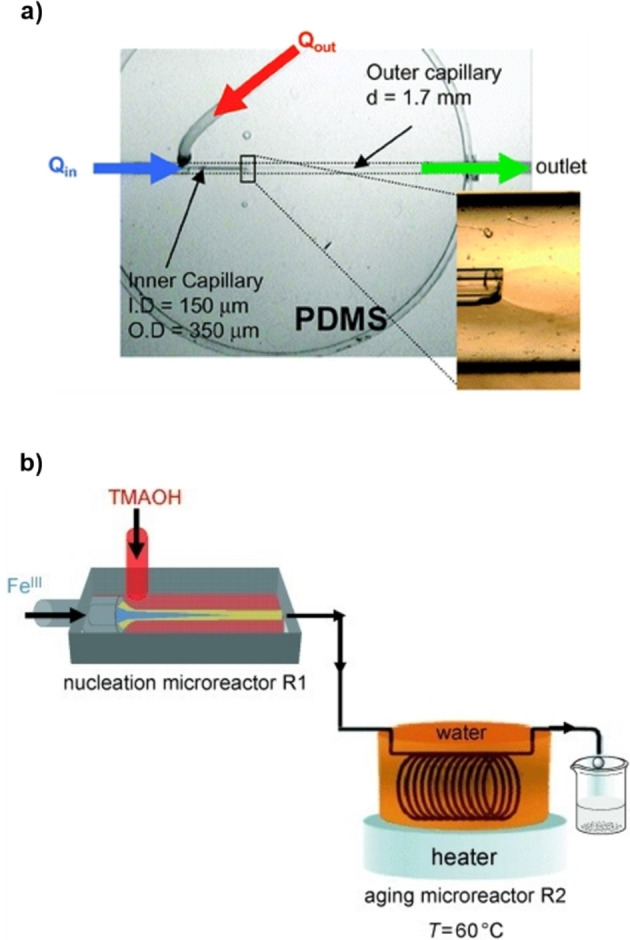
a) Microreactor for 3D‐flow coaxial flow developed by Hassan et al. Reproduced with permission from Ref. [10] Copyright Royal Chemical Society, 2008. b) Schematic representation of the system for nucleation and growth of goethite NPs. Reproduced with permission from Ref. [19], Copyright Wiley‐VCH, 2009.

Hassan et al. reported the synthesis of γ‐Fe_2_O_3_ NPs in a microfluidic device with a 3D coaxial flow setup at room temperature.^[10]^ The Fe(II)/Fe(III) chloride solution was injected through a capillary into a stream of aqueous tetramethylammonium hydroxide (TMAOH, Figure 2a). The rate of the two flows (Q_out_/Q_in_) was adjusted to avoid any turbulence and to slightly defocus the inner stream into the outer stream. The mixing was controlled only by diffusion and a pH gradient was created between the central flow and the channel periphery. This induced the co‐precipitation of NPs in the neutral zone between the two streams, avoiding contacts of the NPs with the channel walls. A few seconds after mixing, the products were poured in a collection vial with a surfactant solution to prevent NPs coalescence. Optimized conditions gave spherical NPs with an average size of 7 nm and size distribution σ=0.20.

The same methodology was applied to the synthesis of goethite (FeOOH) NPs using iron(III) chloride and TMAOH.^[19]^ The microfluidic system consisted in two sections; a compart based on the previously discussed 3D injection strategy for the nucleation process and a PTFE coil reactor in a 60 °C heating bath for the controlled growth of the NPs (Figure 2b). The mixing process was quantitatively analyzed using an acidic fluorescein solution in the inner stream and the TMAOH solution in the outer stream. By probing the pH‐dependent fluorescence intensity at different flow conditions, an optimal mixing time of 80 ms was set for the ratio Q_out_/Q_in_=400. The ferrihydrite NPs obtained outside the 3D coaxial mixer were spherical with a diameter of 4±1 nm. After a controlled growth in a second compartment with a residence time of 15 min, nanoplates with an average length of 30±17 nm and width of 7±4 nm were collected. Notably, the microfluidic system accelerated the goethite nanoplates synthesis that typically requires several hours under batch conditions. The authors proposed that the shear stress derived from the laminar flow in a thin microtubular reactor favors a pre‐alignment of the primary NPs that speeds up their aggregation.

**Figure 3:**
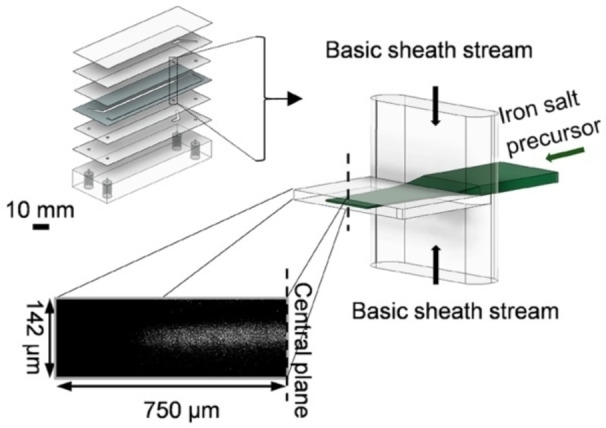
Multilayer microfluidic tape device developed by Seidal et al. The bottom picture illustrates the flow profile obtained by confocal microscopy of a focused fluorescein solution stream used instead of the iron precursors solution. Reprinted (adapted) with permission from Ref. [20], Copyright 2018, American Chemical Society.

In 2018, a 3D flow focusing device was employed by Seidel et al. in an integrated system for synthesis, functionalization, and relaxivity determination of iron oxide NPs with a diameter of ∼20 nm for magnetic resonance imaging (MRI) applications.^[20]^ Conversely to the previous examples, in which the reactor was made of PDMS by soft lithography, the 3D‐flow system was a multilayer device composed by alternating double‐sided, pressure‐sensitive tape and poly(methyl methacrylate) (PMMA) sheets (Figure 3) without using a clean room facility for its fabrication. In the device, the focusing of the acidic metal precursor solution into two sheath streams of aqueous NaOH takes place. The diffusion of the base into the central stream triggered the precipitation of NPs. The pH has a major influence in controlling particle size and, usually, smaller NPs are obtained at high base concentration. By regulating the iron and base concentrations as well as the Q_out_/Q_in_ ratio, it was possible to keep the pH around 10 and synthesize NPs with a mean diameter of 24.6±7.1 nm. Similar pH adjustments are difficult in a batch synthesis without obtaining nonmagnetic impurities.^[21]^

The particles composition was assessed by Raman spectroscopy that showed mostly Fe_3_O_4_, along with some γ‐Fe_2_O_3_ resulting from oxidation at the NPs surface. The authors reported a productivity value of 40 μmol/h^−1^ for magnetite, which could be increased by running the synthesis within several devices in parallel.

Staniland et al.^[22]^ furtherly exploited the coaxial injection system reported by Hassan.^[6]^ A device, made of polyether ethyl ketone (PEEK) with a glass capillary as the reaction vessel, was fabricated and compared with a traditional PDMS‐based reactor. The authors investigated the effect of the Fe^2+^/Fe^3+^ ratio in the precursor solution on the product morphology and composition, finding 0.5–0.6 as the optimal fraction of ferrous ions for the synthesis of magnetite NPs. The synthesis was carried out also in the presence of a protein (magnetite biomineralisation protein, Mms6) which led to slightly smaller NPs showing an increased magnetic saturation.

### 3.3 Droplet‐based Reactors

**Figure 4:**
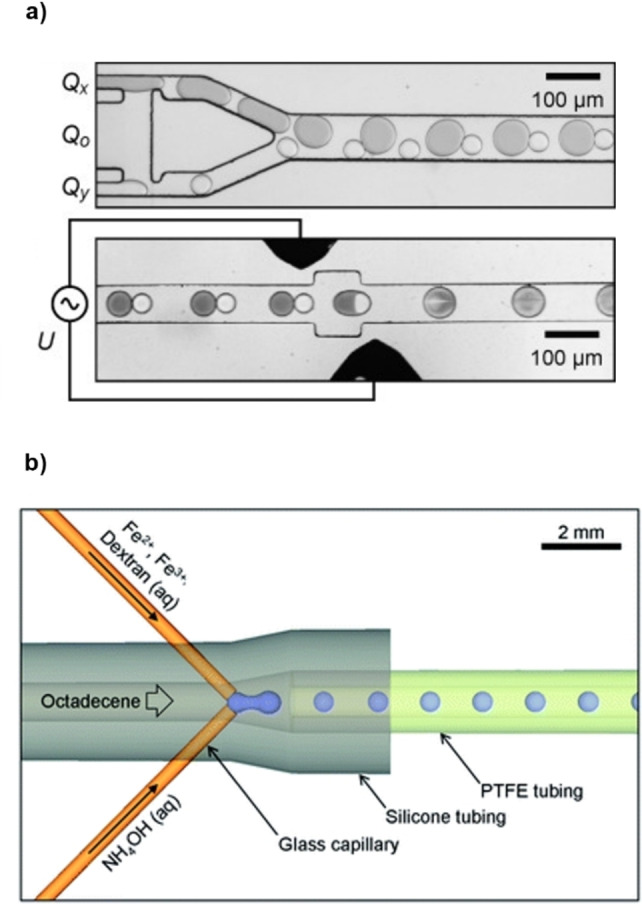
Schematic representation of the microreactors for droplet‐based continuous flow synthesis of iron NPs. a) Module for emulsification and droplet pairing of the iron (Q_X_) and base (Q_Y_) solutions in the oil phase (Q_O_) (up) and module for the electrocoalescence of the droplets pair by application of a voltage U between two electrodes. Reproduced with permission from Ref. [23] Copyright Wiley‐VCH, 2008. b) Capillary‐based droplet reactor for the injection of iron precursors and base in the carrier stream. Reproduced with permission from Ref. [24], Copyright Royal Chemical Society, 2012.

Baret et al.^[23]^ described a system in which the aqueous streams of metal precursors (Fe(II)/Fe(III) chloride 2 : 1) and base (NH_4_OH 2M) were turned into a flow of droplet pairs in a perflurocarbon oil phase with a 2.5 wt% of a perfluorinated surfactant. The reactants were mixed through the electrocoalescence of the droplet pair to form Fe_3_O_4_ NPs. The pairing of metal precursors and base droplets was highly reliable thanks to the synchronous emulsification of the two streams in a single module (Figure 4a). The metal/base stoichiometric modulation was controlled by the reagent flow rate. The coalescence of the droplet pairs occurred with the aid of the electric field applied by two electrodes within the microfluidic chip; this led to the formation of a precipitate inside the droplet in 2 ms. The resulting Fe_3_O_4_ NPs were smaller than those obtained by bulk mixing (4±1 nm vs. 9±3 nm).

The droplet‐based methodology was later investigated by the group of deMello.^[24]^ A solution of ammonium hydroxide and a second one containing the metal precursors in the presence of dextran as a biocompatible surfactant, were injected simultaneously into an octadecene carrier stream (Figure 4b). In the small droplets that formed, NPs of iron oxide precipitated. The authors showed how this droplet‐based methodology avoided fouling of the channels wall, conversely to a corresponding monophasic flow reaction. A further step to control particle growth and crystallization was taken by flowing the droplets stream into a capillary at 60 °C. NPs with a mean diameter of 3.6 nm and a narrow distribution (σ=0.8 nm) were obtained. Selected Area Electron Diffraction (SAED) analysis showed crystalline spinel planes of Fe_3_O_4_ and γ‐Fe_2_O_3_, with minimal impurities from non‐superparamagnetic species, such as α‐Fe_2_O_3_. The dextran coating, detected by FTIR analysis, enhanced the stability of the NPs dispersions, preventing aggregation over several weeks.

A droplet‐based methodology with a similar setup to Figure 4b was reported by Chung and co‐workers.^[25]^ The carrier fluid was mineral oil containing a surfactant in a 1.8 vol % amount. The NH_4_OH and FeCl_2_/FeCl_3_ aqueous solutions were injected into the carrier stream through converging capillaries to generate a droplet flow which passed through a heated coil reactor (70 °C). NPs of similar size (10.5 nm in flow vs. 10.6 nm in batch) but worse size dispersion (1.8 nm in flow vs. 2.4 nm in batch) were obtained under similar reaction conditions. Nevertheless, the higher reproducibility coupled with the possibility to control the mean NPs diameter (5–12 nm) by tuning temperature (40–90 °C) and residence time (2–20 min), makes the flow approach a convenient alternative to the batch method.

Another approach based on droplet pairing and passive coalescence was recently applied to a microfluidic system able to endure long reaction times (up to 48 hours) thanks to a drainage system that greatly reduces the risk of clogging.^[26]^ The microreactor was made of a PMMA channels sheet between two cover plates. The FeCl_2_/FeCl_3_ precursors and the base (NaOH) were injected in two separate T‐junctions in a carrier stream of hexadecane in the presence of a surfactant (10 wt% amount). A precise pairing of the droplets of the two reactants was obtained by tuning the flow rates, so that a drop of the base solution generated at the first T‐junction collided with a forming droplet of the iron salts solution at the second T‐junction. The droplets pair, dragged by the carrier stream, collapsed spontaneously while flowing in a spiral‐shaped microchannel. Precipitation occurred in about 4 ms after the droplets fusion and magnetite NPs were collected at the microreactor outlet with an average size of 7 nm.

### 3.4 Other Microfluidic Strategies

**Figure 5:**
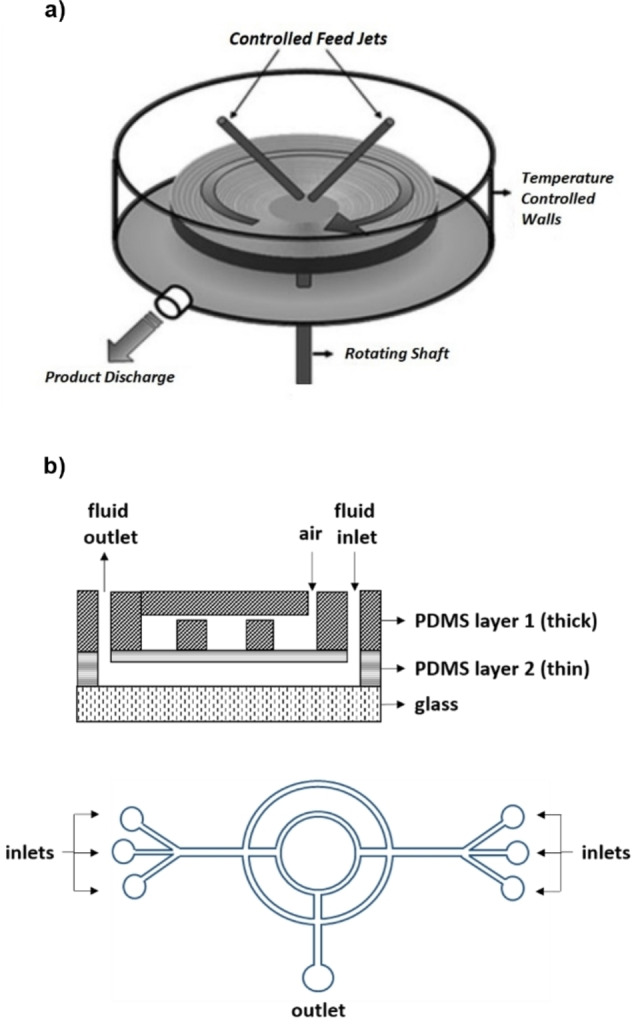
a) The SDP device described for continuous flow synthesis of superparamagnetic NPs. Reproduced with permission from Ref. [27], Copyright 2008 WILEY‐VCH Verlag GmbH & Co. KGaA, Weinheim. b) Schematic representation of the microreactor for the active mixing of the iron precursors and base solutions employed in Ref. [28]. (Up) Cross‐sectional view, PDMS layer 1 is employed as the air channel, PDMS layer 2 is employed as the fluid channel. (Down) Top view of PDMS layer 2 showing the double‐loop micromixer structure.

A continuous flow synthesis of magnetite NPs was reported with a flow spinning disc processing (SDP).^[27]^ The apparatus consisted of a rotating disc (up to 3000 rpm) with two feed jets above the disc center. The reagents solutions were deposited over the rapidly rotating system and dragged towards the disc borders by centrifugal forces (Figure 5b). The resulting thin and turbulent liquid film enables fast mixing and mass/heat transfer. The feed jets carried a FeCl_2_/FeCl_3_ aqueous solution and the reactor chamber was filled with gaseous NH_3_. Ammonia was chosen instead of aqueous NH_4_OH to better control the nucleation and growth of magnetite NPs. The reaction with ammonium hydroxide in solution is very fast and the particle size is independent on the operating parameters. However, by using gaseous ammonia, the disc rotating speed can control the base transfer into the liquid film. For instance, at 2500 rpm, irregular ultrafine NPs (3–5 nm) were obtained, as a high rotation ensures a wave regime on the fluid film and consequently a greater gas absorption and more NPs nucleation points. At 500 rpm, spherical particles with a 10 nm diameter were obtained since the NH_3_ absorption is highly reduced and the growth process becomes dominant.

Lee et al. provided an interesting example of a sophisticated microreactor chip for iron oxide NPs synthesis^[28]^ consisting of a double‐loop micromixer equipped with two micropumps and a microvalve for the active mixing of the reagents. The chip is composed by a cover glass with a double layer of PDMS (Figure 5a). The top layer is equipped with air inlets, the middle PDMS layer, corresponding to the ceiling of the fluid channel, is thin and can be pneumatically deflected by applying a pressure from the air microchannel above. This operation originates a peristaltic‐like behavior that triggers the fluid motion. Thanks to a microvalve that electronically controls the air flow, the solution can be transported clockwise or counter‐clockwise for a more effective mixing. The authors provided a test in which, with an air pressure of 15 psi and a driving frequency of 7.7 Hz, the complete mixing of the two solutions was achieved in just 2 s. The iron NPs synthesis was managed as follows; as iron(II) chloride and iron(III) chloride acidic solution were transported from their reservoirs and vigorously mixed, a NaOH solution was added and mixed until “brownish‐black” colors were observed as a sign of NPs formation. The whole process required 15 min and led to the isolation of iron NPs in the size range of 4.8–6.7 nm, depending on the reagent ratio and with a narrow distribution. Noteworthy, this system gives the possibility to work in the absence of additives and with high reagent concentrations (1–2 M for iron chloride solutions), although in very small volumes and not in continuous flow.

The possibility to perform in‐line analysis with ease is one of the benefits given by flow technologies. Gavriilidis et al. exploited this opportunity to gain mechanistic insights in the co‐precipitation and stabilization of iron oxide NPs and develop an efficient continuous flow synthesis.^[29]^ The mechanism of NPs formation during co‐precipitation is still unclear because of the process high rate; however, with a flow chemistry apparatus it is possible to “freeze” the reaction at steady‐state conditions by placing a flow cell for spectroscopic analysis at a determined distance from the micromixer. The chemical composition at different reaction times can be thus investigated by modulating the flow rate. With in situ synchrotron X‐ray diffraction (XRD) analysis, the authors demonstrated the formation of crystalline maghemite/magnetite NPs 5 s after reagents mixing, standing in contrast to previous mechanistic studies claiming the presence of an intermediate crystalline phase slowly converting into magnetite.^[30]^ The agglomeration of the iron oxide NPs obtained by mixing the 2 : 1 FeCl_3_/FeCl_2_ aqueous solution with NaOH 2 M was assessed through in situ small angle X‐ray scattering (SAXS). This revealed highly agglomerated NPs, which had to be de‐agglomerated and stabilized in flow by neutralization with a citric acid solution in a second coil reactor. The system was robust against fouling thanks to the high flow rate (5 ml/min) and to the highly negative zeta potential of PTFE tubing in the presence of alkaline solutions. The system allowed the synthesis of superparamagnetic NPs in the 6–7 nm size and in a promising amount for large‐scale applications (1.5 mg/mL NPs solution produced at a 500 mL/h flow rate).

## 4 Silica Nanoparticles

Silicon oxide nanostructures feature an enormous range of applications in medicinal chemistry,^[31]^ (photo)catalysis,^[32]^ molecular electronics^[33]^ and sensing,^[34]^ to name a few. Their properties are closely related to their size and shape, usually tuned by using specific synthetic conditions. The sol‐gel process, which is generally based on the hydrolysis and condensation of silicon alkoxides, is a common strategy to prepare silica nanoparticles (SNPs) as it is a simple, low‐cost and low‐temperature method to obtain highly dispersed materials. A first example was reported by Stober^[35]^ in 1968 by using TEOS (tetraethyl orthosilicate) as the silicon precursor and NH_3_ as a base. Continuous flow approaches are well‐suited to the synthesis of SNPs for the high control that one can achieve on heat, mass transfer and other experimental parameters that greatly influence the quality of the final SNPs, achieving the results summarized in Table 2.

**Table 2:** Silica NPs syntheses in continuous flow.

Oxide	Reactants	Methodology	Year	T	Shape	Size^[a]^ [nm]	Size distribution [σ_d_/d]	Ref.
SiO_2_	TEOS +NH_3_/H_2_O	segmented flow	2004	RT	spheres	277^[b]^	0.095^[b]^	[36]
SiO_2_	TEOS/APTES+NH_3_/H_2_O	reactor with interdigital micromixer	2012	40 °C	spheres	79	0.24	[37]
SiO_2_	TEOS/APTES+NH_3_/H_2_O	droplet‐based reactor	2012	RT	spheres	350^[b]^	0.03^[b]^	[38]
SiO_2_	Na_2_SiO_3_+H_2_SO_4_	membrane dispersion mixer	2014	RT	–	18–21^[c]^	–	[42]
SiO_2_/TiO_2_ (STNPs)	TTIP+H_2_O TEOS+NH_3_/H_2_O	multistep nucleation‐controlled growth	2015	RT	–	33	0.14	[39]
SiO_2_ (HSSs)	TEOS+NH_3_/H_2_O	spiral microreactor	2017	RT	hollow spheres	805 (20 nm shell thickness)	0.14	[40]

[a] Calculated by TEM/SEM analysis. [b] The example with narrower size distribution is reported. Different sizes were obtained by modulating the flow rates. [c] SNPs size range obtained by changing the reaction parameters.

### 4.1 Sol‐gel processing in multiphase systems

In a 2004 seminal work, Jensen et al. investigated the synthesis of colloidal SNPs in a microreactor^[36]^ using TEOS and NH_3_/H_2_O in ethanol. A single‐phase laminar flow reactor (LFR) or a segmented flow reactor (SFR) equipped with a gas inlet to create an air/liquid slug flow were used to grow the NPs. Those obtained with the LFR suffered of a poorer size distribution, if compared to batch synthesis. This was attributed to axial dispersion effects induced by the slow motion of particles close to the channels wall with respect to those in the flow center. This difference leads to a larger residence time distribution and therefore to a poorer size homogeneity of the final NPs. This effect is particularly evident at high flow rates, when low residence times and smaller NPs are needed. The size distribution issue was overcome with SFRs where the fluid flow is divided into a number of small comparts by the intercalation of air. Each compart is equivalent to a small batch reactor in which microcirculation occurs. In this way, the SNPs size distribution was comparable to that observed in batch syntheses. The gas inlet that produces the slug flow was placed after the reagents mixing region, allowing a uniform reagent distribution in the thin films formed between adjacent liquid segments (Figure 1b) and preventing an additional cause of size dispersion.

**Figure 6:**
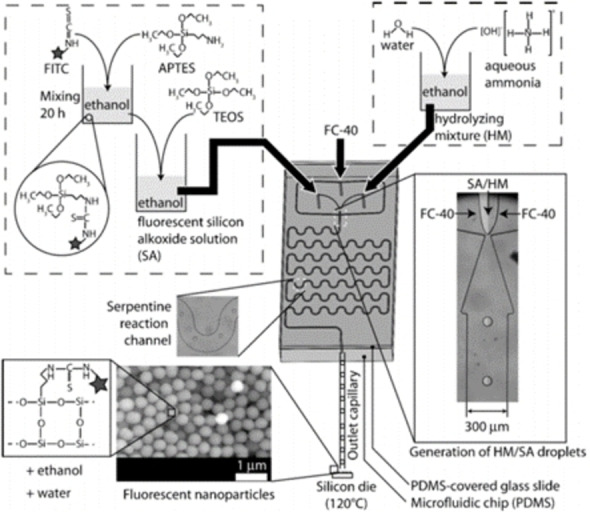
Schematic procedure for fluorescent SNPs synthesis. The bottom right picture illustrates a close‐up of the flow‐focusing nozzle in which the droplet stream in the fluorinated carrier (FC‐40) is formed. Reproduced with permission from Ref. [37], Copyright Royal Chemical Society, 2012.

A different droplet‐based approach was used to prepare fluorescent SNPs.^[37]^ in which ethanol droplets, containing TEOS and aminopropyl triethoxysilane (APTES) functionalized with fluoresceine, were carried by an immiscible phase of a perfluorinated hydrocarbon. In a PDMS microfluidic chip, the silicon alkoxides droplets stream (SA) merged with the NH_3_/H_2_O hydrolyzing mixture (HM), through a flow‐focusing nozzle (Figure 6). The SNPs nucleation/growth process occurred inside the droplet while the carrier ran through a serpentine. The mixture was collected on a heated silicon chip where the liquid immediately evaporated. The particles average diameter, in the range of 50‐350 nm, mainly depends on the flow rates, whereas their uniformity on HM/SA ratios and residence time. An exceptionally low size dispersion of 3 % was found in one case. Interestingly, the particles growth rate was remarkably higher in this droplet‐based synthesis, if compared to the batch one.

### 4.2 Sol‐gel process in homogeneous systems

A microfluidic methodology was employed by Santamaria et al. for the synthesis of SNPs to be used for SiO_2_‐Au core‐shell plasmonic structures.^[38]^ Amino‐functionalized SNPs were obtained at 40 °C using ethanol as solvent. TEOS and APTES were efficiently mixed with an NH_4_OH solution inside a commercial slit interdigital micromixer that splits and recombines the reagent flows. The mixture is then passed through a PTFE tube where the SNPs growth takes place, reaching an average size of 79±19 nm. The size distribution is quite scattered probably because a mono‐phasic laminar flow was adopted, thus leading to the previously discussed axial dispersion effect.

**Figure 7:**
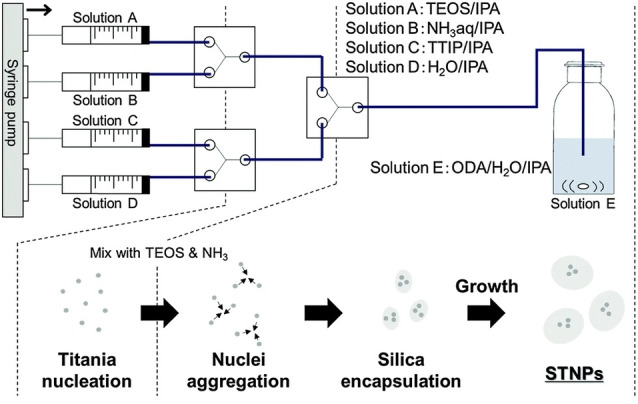
mNCS system for the synthesis of hybrid STNPs. Reproduced with permission from Ref. [39], Copyright Royal Chemical Society, 2015.

The multistep nucleation‐controlled growth (mNCS) is a method to synthesize hybrid core‐shell NPs with uniform and tunable size developed by Shiba et al.^[39]^ They reported a study in which pre‐formed, well‐defined titania seeds are employed for the growth of hybrid silica NPs. In the relatively simple microfluidic setup, titanium tetraisopropoxide (TTIP) and TEOS were reacted separately with a hydrolyzing solution (Figure 7) and the two resulting streams were subsequently mixed.

The faster hydrolysis of the titanium alkoxides led to the formation of small TiO_2_ aggregates at the second Y‐mixer while TEOS was nearly unreacted. The titania nuclei were then encapsulated by silica, yielding silica‐titania nanoparticles (STNPs) with an average size of 33 nm with a coefficient of variation of 0.14. The corresponding synthesis in the absence of titania yielded monodispersed silica spheres with a large 550 nm diameter, as an evidence of the control given by mNCS methodology. In principle, the same approach can be used for the controlled synthesis of several core‐shell hybrid NPs containing titania.

**Figure 8:**
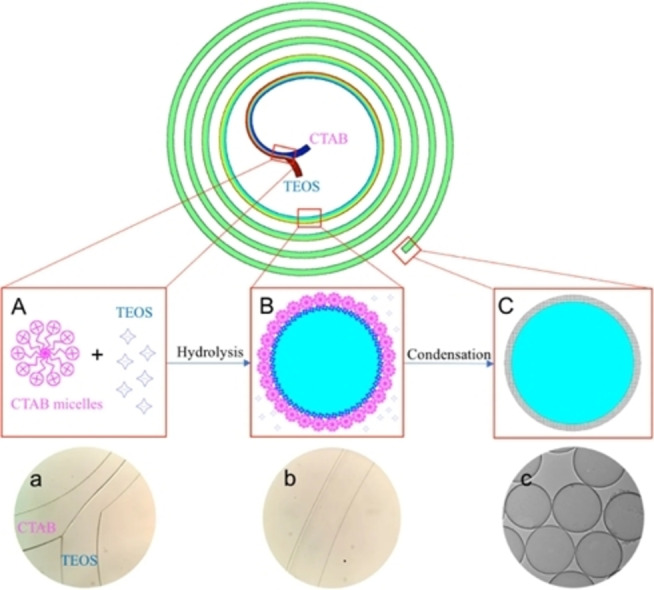
Representation of the spiral microreactor. (A) and (a) interphase between the aqueous CTAB solution and ethanolic TEOS solution where the TEOS hydrolysis occurs. (B) and (b) a shell layer derived from the condensation of hydrolyzed TEOS is formed after complete mixing and it is stabilized by CTAB micelles. (C) and (c) further condensation over the shell layer originates the HSSs collected at the outlet. Reproduced from Ref. [40] under CC BY 4.0, Copyright The Authors, 2017.

An interesting strategy to prepare hollow silica spheres (HSSs) in the sub‐micrometer range was reported by Zhang and coworkers.^[40]^ The microfluidic system was a spiral microchannel that exploited the transverse Dean flow (Figure 1e) for effective mixing of an ethanol solution containing TEOS and an aqueous solution of ammonia and hexadecyltrimethylammonium bromide (CTAB). The Dean vortices, triggered by keeping the flow rate at 400 μl/min in the curved channels, led to a very fast mixing and production of HSSs in less than one second. In the proposed mechanism, shown in Figure 8, the NH_3_‐catalyzed hydrolysis of TEOS at the flows interphase, leads to spherical micelles that undergo a fast condensation, affording the HSSs with an average size of 805±111 nm with a shell thickness of ∼20 nm.

Mesoporous silica nanofibers were obtained, instead of HSSs, with a slower flow rate of 10 and 100 μl/min for TEOS and CTAB/ammonia solutions respectively, in a similarly curved microreactor.^[41]^

### 4.3 Other microfluidic methodologies

Highly dispersed SNPs^[42]^ were produced in a membrane microreactor by reacting sodium silicate (Na_2_SiO_3_) with sulfuric acid in the presence of a surfactant that limits particles polymerization through superficial silanol groups. An efficient mixing of the reagents was obtained by pumping the H_2_SO_4_ aqueous solution through a 5 μm stainless steel membrane into a continuous flow of a Na_2_SiO_3_/surfactant solution in water. The so‐obtained slurry was collected in a reservoir, the pH was adjusted to the value of 9, then the particles were isolated after water/ethanol washing and drying. Primary particles with a mean size of 20 nm were characterized by TEM analysis. Their aggregation was evaluated through the dispersion index *D_1_* defined as $D_{1}=S_{C}\;/S_{BET}$ where *S_C_* is the theoretical surface area considering monodispersed particles with the diameter observed by TEM characterization and *S_BET_* is the experimental value obtained by BET surface area analysis. A *D_1_* of 1.12 was obtained under the optimized flow speed, final pH and surfactant amount, reflecting a limited aggregation of the primary particles by this methodology.

## 5 Zinc Oxide Nanoparticles

Zinc oxide in the nano size range is a widely employed semiconductor with a wide band gap (3.37 eV) and a large exciton binding energy (60 eV). ZnO NPs are inexpensive and nontoxic particles that exhibit remarkable optical and electrical properties for applications in materials science and biomedical fields.^[43]^

It has been reported that microfluidic technologies, applied to solution‐based syntheses of ZnO NPs, help to control and improve their quality and characteristics (as reported in Table 3).

**Table 3:** Zinc‐ NPs syntheses in continuous flow.

Oxide	Reactants	Methodology	Year	T	Shape	Size^[a]^ [nm]	Size distribution [σ_d_/d]	Ref.
ZnO	ZnCl_2_+NH_4_(CO_2_NH_2_)	FIS	1999	RT	rods	10–15^[b]^	–	[44]
ZnO	Zn(AcO)_2_+NaOH	microreactor chip	2014	60 °C	spherical	3–5	–	[45]
ZnO	Zn(AcO)_2_+TMAOH (w/an organic acid)	convective tangential micromixer+coil reactor	2014	20–80 °C	–	3.7^[c]^	0.11^[c]^	[51]
ZnO	Zn(NO_3_)_2_+NaOH (microemulsions)	micromixer+coil reactor	2014	40–70 °C	–	10–20^[d]^	–	[49]
ZnO	Zn(NO_3_)_2_+NaOH	microreactor chips	2017	60 °C	spheres	10 nm	–	[46]
					spindles	300–600 nm	–	
ZnO	Zn(AcO)_2_+NaOH	droplet‐based mircroreactor	2017		spherical	49.6	0.16	[50]
ZnO	Zn(NO_3_)_2_+NaOH	spiral microreactor	2019	RT	spheres	583	0.21	[47]
					cubes	153	0.24

[a] Calculated by TEM/SEM analysis. [b] Diameter. [c] At 20 °C. [d] The NPs size depended on the employed conditions (flow rate, T, zinc concentration) fairly monodispersed NPs were obtained in single runs.

### 5.1 Flow injection synthesis

An early example of ZnO NPs synthesized under continuous flow conditions was reported in 1999 by Mamoun et al. using the Flow Injection Synthesis strategy.^[44]^ Solutions of ZnCl_2_ and ammonium carbamate were injected in two separated carrier streams forming two regularly segmented flows. By pumping at the same rate through tubes of the same length, the zinc and carbamate segments merged simultaneously in a T mixer, producing the precipitation of Zn_5_(OH)_6_(CO_3_)_2_ that, upon calcination at 350 °C, gave pure ZnO nanorods with a diameter of 20 nm.

### 5.2 Continuous flow synthesis in microreactor chips

**Figure 9:**
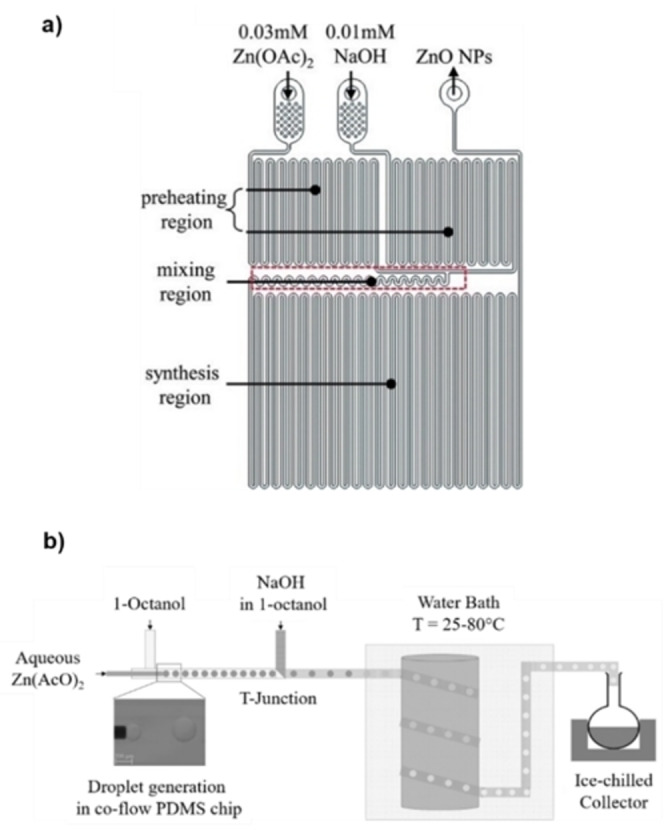
a) Schematic diagram of the chip reactor used for ZnO NPs synthesis by Sung et al. Reproduced with permission from Ref. [45] Copyright Royal Chemical Society, 2014. b) Experimental setup for the droplet‐based synthesis of Zn NPs. Reprinted (adapted) with permission from Ref. [49], Copyright 2017 American Chemical Society.

Sung and co‐workers tackled in 2014 the synthesis of ZnO NPs in a microreactor, equipped with a preheating zone and a mixing area (Figure 9a), by treating Zn(AcO)_2_ and NaOH in ethanol at 60 °C.^[45]^ A pulsed instead of a continuous flow was applied to move the reagents. Theoretical simulations helped to optimize the pulse frequency for an effective mixing and for having a continuous stream at reactor outlet. A dispersion in ethanol of ZnO NPs, with a size of 3–5 nm, was collected after a residence time of 35 s. This size was almost identical to that of NPs prepared in batch with the same reagents but after 2 h reaction time. Another distinctive difference between batch vs. flow approaches was the stability of the NPs dispersion prepared in flow that showed no flocculation for over 14 days, if compared to that prepared in batch, as confirmed by zeta potential measurements.

In a recent report^[46]^ the influence of the microreactor geometry on the final ZnO NPs characteristics was investigated by Ganguli and co‐workers. Four PDMS chips with different depth, width, and length of the microchannel network were employed to react Zn(NO_3_)_2_ with NaOH in water at 60 °C, yielding a variety of ZnO nanostructures with different morphologies, such as spheres, spindles or nanosheets, respectively. To explain these results, the authors associate the observed NPs morphology with the ability of each microreactor geometry to manage heat transfer during the NPs synthesis. Hydrolysis of Zn(NO_3_)_2_ leads to Zn(OH)_2_ in a fast and diffusion‐controlled process; its temperature‐dependent conversion to ZnO is the rate determining step. Different reactor geometries originated different temperature gradients that led to the observed particle diversity.

The synthesis of ZnO NPs was also reported by Zhang et al. in a spiral‐shaped microfluidic chip.^[47]^ Interestingly, different particle shapes, that is, spheres, ellipsoid, rods, cubes, urchin‐like, and plates, were obtained by varying the flow rate of the Zn(NO_3_)_2_ and/or NaOH solutions. However, only cubic and spherical particles fitted in the size range of nanoparticles (153±36 nm and 583±124 nm respectively). All these ZnO particles were tested as photocatalysts for dyes photodegradation, cytotoxic agents and piezoelectric materials. The NPs with the largest surface area (cubic and urchin‐like) performed nicely in all three applications.

### 5.3 Continuous flow synthesis in multi‐phase systems

The microemulsion approach for NPs synthesis is a popular strategy that avoids excessive particle growth by confining the reagents in small droplets.^[48]^ A combination of microemulsions with microfluidics was investigated by Zhao et al. in an experiment where inverse microemulsions of Zn(NO_3_)_2_ and NaOH in octane with CTAB/n‐BuOH as surfactants,^[49]^ flowed through a micromixer and stainless steel tubings at 50 °C. ZnO NPs with a smaller average size (16 nm) and a better size dispersion, than those of NPs prepared in batch, were obtained after calcination at 350 °C.

A droplet‐based methodology was reported by Gupta et al.^[50]^ to prepare ZnO NPs. Droplets of Zn(AcO)_2_ in a water/octanol phase were mixed with a stream of NaOH in octanol at 25–80 °C (Figure 9b). The NPs formation was driven by diffusion of the base into the droplets containing zinc acetate. The crucial event for NPs nucleation and growth is mass transfer of the base which is highly dependent on droplet size, temperature, and reagent concentrations; all parameters that can be readily investigated and optimized in a continuous flow reactor. It has been found that morphology was very sensitive to temperature: spherical NPs, for instance, were produced at 60–80 °C, with an average size of 41–62 nm. This work showed that more homogeneous and less size‐dispersed ZnO NPs could be obtained with a droplet‐based approach at relatively low temperatures, if compared to single phase, batch reactions.

### 5.4 Continuous flow synthesis of ZnO quantum dots

Microfluidic technology enabled the synthesis of ZnO quantum dots^[51]^ exhibiting photoluminescence with a quantum yield up to 30 % in the visible range. A solution of Zn(AcO)_2_ and an organic acid in ethanol was mixed, through a miniature convective tangential micromixer, with a solution of tetramethyl ammonium hydroxide in ethanol and flowed through a heated serpentine. Dots with a 4‐5±0.4–0.6 nm diameter were obtained at a flow rate of 4.5 mL/min in the temperature range of 20–80 °C and a productivity of 3 g/h, using propionic acid as a capping agent to avoid aggregation.

## 6 Titania Nanoparticles

TiO_2_ NPs have outstanding electronic and optical properties that make this material one of the most studied inorganic semiconductors^[52]^ with extensive applications in solar energy conversion and photocatalysis,^[53]^ where it is especially important for pollutants removal from air and water, to name a few. Sol‐gel methodologies for TiO_2_ NPs synthesis are attractive for low processing temperatures (<100 °C) and control over particle size and morphology. However, sol‐gel methods afford poorly crystalline materials which must undergo calcination to afford a functional product. This thermal process often causes NPs aggregation, thus increasing NPs size and reducing the active surface area. A number of microfluidic, low temperature, sol‐gel methods are presented herein (Table 4). They involve the hydrolysis of a titanium alkoxide to produce fine uniformly sized TiO_2_ NPs with enhanced properties with respect to the bulk synthesis under comparable conditions. Although this review focuses on low temperature flow approaches, it is worth mentioning that several continuous flow methods at high temperatures were also reported to enhance the crystallinity of the final material.^[54]^

**Table 4:** Titania NPs syntheses in continuous flow.

Oxide	Reactants	Methodology	Year	T	Shape	Size^[a]^ (nm)	Size distribution (σ_d_/d)	Ref.
TiO_2_	TTIP+H_2_O	ceramic microreactor chip	2002	RT	–	<10	–	[55]
TiO_2_	TTIP+H_2_O	axle dual pipe device (3D‐flow injection)	2004	RT	spherical	40–150^[b]^	–	[11]
TiO_2_	TTIP+H_2_O	droplet‐based reactor	2018	RT	spherical	8–10	–	[56]

[a] Calculated by TEM/SEM analysis. [b] Particles size depended on the inner channel width, fairly monodispersed NPs were obtained in single runs.

### 6.1 Titania nanoparticles synthesis

**Figure 10:**
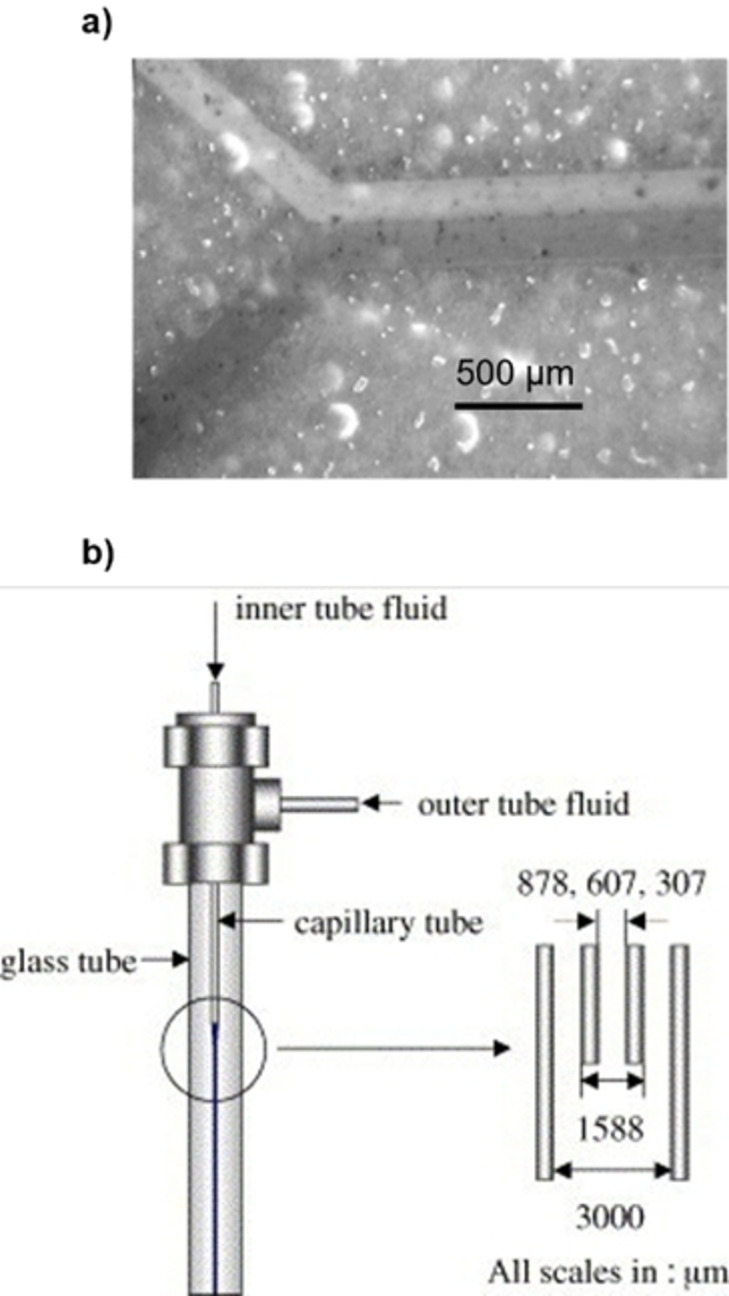
a) Close‐up of the Y‐mixer in the ceramic chip where a stable interphase between water (red ink was added) and cyclohexane is formed. Reproduced with permission from Ref. [55] Copyright Royal Chemical Society, 2002. b) Schematic representation of the axle dual pipe microdevice developed by Mae et al. Reproduced with permission from Ref. [11], Copyright Elsevier, 2003.

Among the first examples of TiO_2_ NPs synthesis in a microfluidic device at room temperature, Maeda et al.^[55]^ reported the formation of titania particles through the reaction between TTIP and water at the interface of two immiscible streams (Figure 10a). The reaction was carried out in a ceramic chip in which a polar solvent (water or a mixture water/formamide) was injected along with TTIP dissolved in cyclohexane or 1‐hexanol. The two phases merged in a Y‐junction and the flow rates were regulated to create a stable interface where the TiO_2_ colloidal NPs grew. The NPs, collected at the outlet, had a size <10 nm as detected by TEM analysis.

The same approach was further developed by Mae and co‐workers with a microdevice composed by two coaxial pipes to create an annular flow of two immiscible phases (Figure 10b).^[11]^ The outer phase was an isopropanol/water mixture while the inner stream was composed by a 1 % TTIP solution in cyclohexane, 1‐hexanol, 1‐octanol or 1‐decanol. By this approach, the nucleation occurred only at the interface and no deposition was observed on the reactor walls. The velocities of the inner and outer flow were regulated to form a stable annular flow and the influence of the inner flow width on the resulting NPs was investigated by changing the diameter of the pipe. In particular, the NPs size increased with the inner pipe diameter. A comparison between the flow and the batch synthesis revealed that the isolated NPs had the same XRD signals, but the size distribution was noticeably enhanced with the dual axle pipe system in flow.

**Figure 11:**
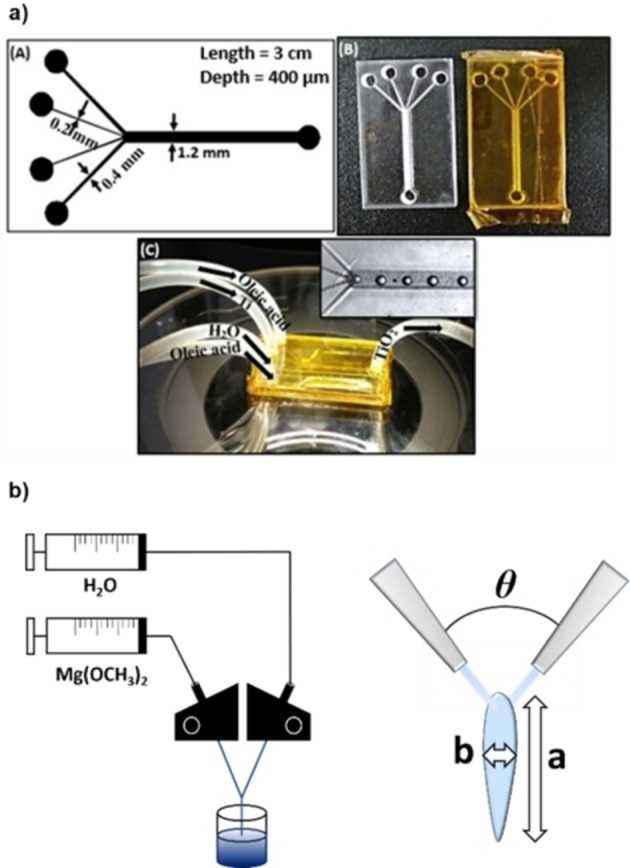
a) Schematic design and digital pictures of the chip reactor employed in Ref. [56]. Reproduced with permission from Ref. [56], Copyright IOP Publishing, 2018. b) Schematic of the system containing the IJM (left) and representation of the mixing zone.

A droplet‐based strategy on a chip was recently developed by Ganguli et al.^[56]^ The microreactor chip was composed of four converging inlet channels; the outer ones carried pure oleic acid and through the inner ones, two streams of TTIP in water and ethanol respectively were introduced separately (Figure 11a). Channels width and flow rates were set to create a focused flow of uniformly sized droplets in the oleic acid stream in which TTIP and water reacted to form the titania NPs. A comparison between batch and flow syntheses, under similar conditions, showed a major difference in NPs diameter: 8–10 nm in flow versus 100–400 nm in batch. XRD analysis of the calcinated samples showed anatase crystals, with traces of orthorhombic impurities only for the batch sample. The authors employed the TiO_2_ NPs obtained in flow as immobilized catalyst on a PDMS microfluidic chip for methylene blue photodegradation.

## 7 Other Metal Oxide Nanoparticles

Herein we present a brief selection of examples for the continuous flow synthesis of NPs composed by other metal oxides, which show particularly effective technological solutions to prevent fouling and clogging phenomena (Table 5).

**Table 5:** Miscellaneous oxide NPs syntheses in continuous flow.

Oxide	Reactants	Methodology	Year	T	Shape	Size^[a]^ (nm)	Size distribution (σ_d_/d)	Ref.
ceria	Ce(NO_3_)_3_+NH_4_OH	membrane dispersion	2017	20 °C	spherical	7.5	0.09	[60]
MgO	Mg(OCH_3_)_2_+H_2_O	impingement jet micromixer	2013	RT	nanocrystalline material	–	–	[65]
ZrO_2_	Zr(O^t^Bu)+H_2_O	axle dual pipe device (3D‐flow injection)	2013	RT	spherical	100–600^[b]^	0.2–0.3	[12]
Cu_2_O	Cu	vortex fluidic device	2019	RT		14^[c]^	0.07^[c]^	[69]
CuO		for pulsed laser ablation				11^[c]^	0.09^[c]^

[a] Calculated by TEM/SEM analysis. [b] Size range obtained by modulating residence times and reagents concentration in the absence of surfactant. [c] Determined by the Scherrer equation.

### 7.1 Ceria nanoparticles synthesis

Cerium dioxide (CeO_2_, ceria) has relevant applications as a redox catalyst^[57]^ and as an antioxidant in the biomedical field.^[58]^ Ceria exhibits efficient Ce^4+^/Ce^3+^ redox cycling at the surface and the ability to absorb/desorb oxygen. Morphology plays a central role on the catalytic efficiency of CeO_2_ materials, for example in the oxidation of CO.^[59]^ Therefore, synthetic routes able to control the characteristics of the final nanocrystalline material are highly needed. Wang and co‐workers reported a membrane dispersion microreactor to produce size‐controlled ceria NPs, after calcination, from the room temperature reaction of Ce(NO_3_)_3_ with aqueous ammonia.^[60]^ The latter basic solution was pressed through the micropores of a stainless steel membrane into a stream of a Ce(NO_3_)_3_ water solution. Ceria NPs with a diameter of 7.5±0.7 nm were obtained under optimized conditions. The batch preparation in a stirred tank reactor, under similar conditions, gave ceria NPs with a diameter of 15.3±1.2 nm. The better size dispersion of the particles obtained in flow translated in an enhanced catalytic activity in model oxidation reactions.

### 7.2 Nanostructured MgO synthesis

Magnesium oxide NPs are mainly studied as highly biocompatible antimicrobial agents^[61]^ and as adsorbent for toxic materials.^[62]^ MgO materials are usually synthesized by solution methods such as by precipitation^[63]^ or sol‐gel synthesis.^[64]^ For the latter approach, the change from batch to flow synthesis is hampered by the very fast gelation kinetic of the alkoxide precursor Mg(OCH_3_)_2_ in the presence of water. However, it turned out that the gelation step could benefit from the rapid mixing that one can achieve in a suitably designed mixer under continuous flow conditions. Nanocrystalline MgO was in fact synthesized in flow using an Impingement Jet Micromixer (IJM) where two streams of reagents collided and mixed in a free space (Figure 11).^[65]^ The angle *θ* between the impinging streams can be modulated to have, in the free space outside the tubing, an optimal fluid sheet in terms of thickness and aspect ratio (Figure 11b) without flow fragmentation. A solution of Mg(OCH_3_)_2_ and water were pumped at around 15 ml/min; at *θ*=120° a wide and thin liquid sheet was formed in which mixing between the magnesium alkoxide and water occurs. Thermal treatment of the gel collected after the IJM gave nanocrystalline MgO with a surface area of 340 m^2^/g. An analogue batch procedure yielded a less porous material with a surface area of 190 m^2^/g.

### 7.3 Zirconia nanoparticles synthesis

Zirconium dioxide (ZrO_2_, zirconia) is an important material which finds a number of applications, for example, in fuel cells,^[66]^ in catalysis^[67]^ or as a thermal barrier coating.^[68]^ A microfluidic synthesis of zirconia NPs was performed using the axle dual pipe microreactor showed in Figure 9b.^[12]^ Compared to titania, zirconia formation through alkoxides hydrolysis is a much faster process and more difficult to control. In the concentric reactor, the inner stream was composed by a zirconium tetrabutoxide (ZTB) ethanol solution while the outer stream was a water/ethanol mixture. Spherical monodispersed nanoparticles were obtained under optimized conditions. By changing the residence time in axle dual pipe microreactor the particles average size could be modulated between 100–600 nm. The employment of polyethyleneamine as surfactant in a 0.24 wt% amount in the outer stream allowed to limit further the particle growth and to obtain ZrO_2_ NPs with 4 nm diameter.

### 7.4 Copper oxide nanoparticles synthesis

**Figure 12:**
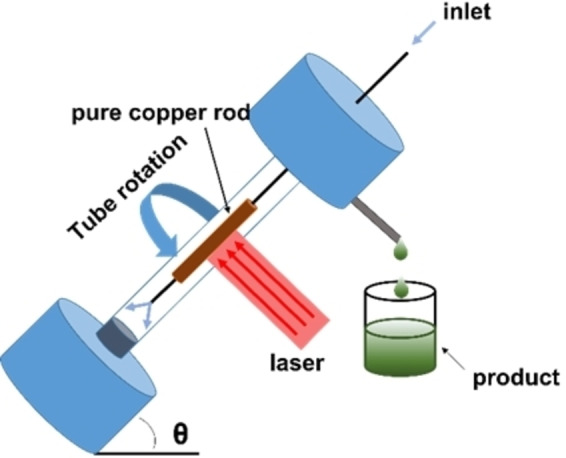
Representation of the VFD device. The copper rod is hosted in a glass tube (20 mm OD, 17.5 mm ID) rotating at high speed (ranging between 4.5 k and 8.5 k rpm) and irradiated with a 5 ns pulsed Nd : YAG laser operating at 1064 nm and 600 mJ.

Copper oxides (CuO and Cu_2_O) NPs find their principal applications in catalysis.^[1g]^ An interesting laser ablation method in flow was developed for the synthesis of copper(I) oxide NPs by Raston et al.^[69]^ The NPs were prepared in a vortex fluidic device (VFD) which consisted in a borosilicate glass tube rotating at high speed (Figure 12). A pure copper rod was inserted in the tube and irradiated with a pulsed laser beam. The plasma plume around the copper rod, in the presence of oxygen, yielded the Cu_2_O NPs. Water was constantly fed through the device inlet; under the centrifugal force the liquid whirled up to the top of the tube and carried the Cu_2_O NPs at the device outlet. The rotation speed and laser power were optimized to minimize the content of copper(II) oxide NPs, yielding Cu_2_O NPs of 14±1 nm. Upon standing in air at 50 °C, Cu_2_O NPs oxidized to CuO NPs of the same size. DLS analysis revealed that, upon standing in solution, both types of NPs gave aggregates with a diameter of around 194 and 91 nm, respectively.

## 8 Conclusions

Flow chemistry is a useful and promising toolbox for the synthesis of metal oxides NPs that may satisfy the need for these important nanosystems in terms of quantity, quality and cost‐effective processing. In this context, we selected a series of contributions that describe the use of continuous flow devices yielding NPs with a precise morphology and limited size dispersion. Engineered flow reactors allow a precise control over experimental parameters and reagent mixing, granting a high reproducibility in the nucleation and growth of metal oxide NPs, if compared to batch approaches. We specifically highlighted technical solutions that minimize product deposition and channel clogging through mixing techniques that avoid the contact of the formed NPs with the reactor walls (e. g., coaxial injection and droplet flow) or through the generation of recirculation motions in the reaction streams (e. g. segmented flow and curvilinear reactors). We hope that this review will help to accelerate further progress in this promising field.

## Conflict of interest

The authors declare no conflict of interest.

## Biographical Information

*Paolo Zardi received his PhD under the supervision of Prof. Emma Gallo at the University of Milan in 2014. He pursued postdoctoral studies at the University of Padova in the group of Prof. Giulia Licini and at the University of Rennes with Dr. Rafael Gramage‐Doria. He currently holds a researcher position at the University of Padova and his interests are focused on the use of microfluidic systems in synthetic chemistry*.

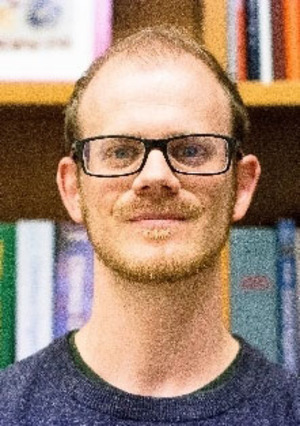

## Biographical Information

*Tommaso Carofiglio received his PhD in 1991 at the University of Lausanne (Switzerland) under the supervision of Prof. Carlo Floriani. Later, he spent one year at the University of Princeton (US) working in Professor J. Groves group on metallo‐porphyrin catalysis. He currently holds an Associate Professor position in Organic Chemistry at the University of Padova. His research interests are focused on the use of microfluidic systems in synthetic organic and inorganic chemistry and on the use of porphyrin‐based receptors for optical sensing*.

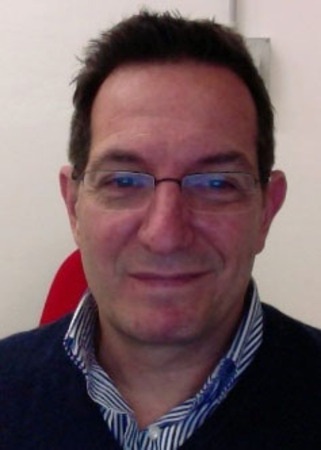

## Biographical Information

*Michele Maggini is full professor of Organic Chemistry and head of the Department of Chemical Sciences at the University of Padova. His research interests focus mainly on the synthesis of molecular organic materials for solar energy conversion and on the development of flow chemistry platforms and methods for the synthesis of functionalized nanosystems, the study of reaction kinetics and of batch‐to‐flow transpositions of distressing or unsafe chemical syntheses*.

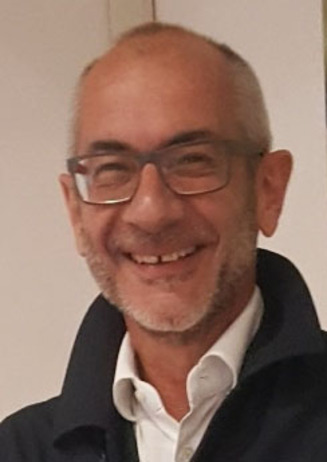

## References

[1a] Y. Zou , B. Huang , L. Cao , Y. Deng , J. Su , Adv. Mater. 2021, 33, 2005215; 10.1002/adma.202005215 33251635

[1b] Q. Liu , Y.-J. Kim , G.-B. Im , J. Zhu , Y. Wu , Y. Liu , S. H. Bhang , Adv. Funct. Mater. 2021, 31, 2008171;

[1c] L. Manna , J. Cheon , R. E. Schaak , Acc. Chem. Res. 2021, 54, 1543–1544; 33820420 10.1021/acs.accounts.1c00123

[1d] C. Gao , F. Lyu , Y. Yin , Chem. Rev. 2021, 121, 834–881; 32585087 10.1021/acs.chemrev.0c00237

[1e] K. K. Paul , P. K. Giri , J. Nanosci. Nanotechnol. 2019, 19, 307–331; 30327040 10.1166/jnn.2019.15778

[1f] R. Mazzaro , A. Vomiero , Adv. Energy Mater. 2018, 8, 1801903;

[1g] C. Ray , T. Pal , J. Mater. Chem. A 2017, 5, 9465–9487;

[1h] N. K. Ojha , G. V. Zyryanov , A. Majee , V. N. Charushin , O. N. Chupakhin , S. Santra , Coord. Chem. Rev. 2017, 353, 1–57;

[1i] R. G. Chaudhuri , S. Paria , Chem. Rev. 2012, 112, 2373–2433. 22204603 10.1021/cr100449n

[2] Z. Wu , S. Yang , W. Wu , Nanoscale 2016, 8, 1237–1259. 26696235 10.1039/c5nr07681a

[3a] C. N. R. Rao , H. S. S. Ramakrishna Matte , R. Voggu , A. Govindaraj , Dalton Trans. 2012, 41, 5089–5120; 22430878 10.1039/c2dt12266a

[3b] A. V. Nikam , B. L. V. Prasad , A. A. Kulkarni , CrystEngComm 2018, 20, 5091–5107;

[3c] H. Tang , J. Wei , F. Liu , B. Qiao , X. Pan , L. Li , J. Liu , J. Wang , T. Zhang , J. Am. Chem. Soc. 2016, 138, 56–59; 26669943 10.1021/jacs.5b11306

[3d] Z. Wang , Y. Ju , S. Tong , H. Zhang , J. Lin , B. Wang , Y. Hou , Nanoscale Horiz. 2018, 3, 624–631. 32254115 10.1039/c8nh00135a

[4] M. B. Plutschack , B. Pieber , K. Gilmore , P. H. Seeberger , Chem. Rev. 2017, 117, 11796–11893. 28570059 10.1021/acs.chemrev.7b00183

[5a] J. Sui , J. Yan , D. Liu , K. Wang , G. Luo , Small 2019, 16, 1902828; 10.1002/smll.201902828 31755221

[5b] L.-J. Pan , J.-W. Tu , H.-T. Ma , Y.-J. Yang , Z.-Q. Tian , D.-W. Pang , Z.-L. Zhang , Lab Chip 2018, 18, 41–56; 10.1039/c7lc00800g 29098217

[5c] G. Niu , A. Ruditskiy , M. Vara , Y. Xia , Chem. Soc. Rev. 2015, 44, 5806–5820; 25757727 10.1039/c5cs00049a

[5d] J. Ma , S. M.-Y. Lee , C. Yi , C.-W. Li , Lab Chip 2017, 17, 209–226. 27991629 10.1039/c6lc01049k

[6a] V. K. LaMer , R. H. Dinegar , Theory , J. Am. Chem. Soc. 1950, 72, 4847–4854;

[6b] C. B. Whitehead , S. Özkar , R. G. Finke , Mater. Adv. 2021, 2, 186–235.

[7a] M. Schoenitz , L. Grundemann , W. Augustin , S. Scholl , Chem. Commun. 2015, 51, 8213–8228; 10.1039/c4cc07849g 25750979

[7b] K. Shahzad , W. V. Aeken , M. Mottaghi , V. K. Kamyab , S. Kuhn , Microfluid. Nanofluid. 2018, 22, 104; 30393471 10.1007/s10404-018-2124-7 PMC6190999

[7c] L. Hartman , Org. Process Res. Dev. 2012, 16, 870–887;

[7d] E. Dressaire , A. Sauret , Soft Matter 2017, 13, 37–48; 10.1039/c6sm01879c 27801463

[7e] H. Wang , A. Mustaffar , A. N. Phan , V. Zivkovic , D. Reay , R. Law , K. Boodhoo , Chem. Eng. Proc. 2017, 118, 78–107.

[8] M. Trojanowicz , K. Kołacińska , Analyst 2016, 141, 2085–2139. 26906258 10.1039/c5an02522b

[9] H. Song , J. D. Tice , R. F. Ismagilov , Angew. Chem. Int. Ed. 2003, 42, 768–772; 10.1002/anie.200390203 12596195

[10] A. Abou Hassan , O. Sandre , V. Cabuil , P. Tabeling , Chem. Commun. 2008, 1783–1785. 10.1039/b719550h 18379692

[11] M. Takagi , T. Maki , M. Miyahara , K. Mae , Chem. Eng. J. 2004, 101, 269–276.

[12] T. Maki , J.-I. Kitada , K. Mae , Chem. Eng. Technol. 2013, 36, 1027–1032.

[13] N. Nivedita , P. Ligrani , I. Papautsky , Sci. Rep. 2017, 7, 44072. 28281579 10.1038/srep44072 PMC5345076

[14] P. Sangaiya , R. Jayaprakash , J. Supercond. Novel Magn. 2018, 31, 3397–3413.

[15] Y. S. Kang , S. Risbud , J. F. Rabolt , P. Stroeve , Chem. Mater. 1996, 8, 2209–2211.

[16a] T. Hyeon , S. S. Lee , J. Park , Y. Chung , H. B. Na , J. Am. Chem. Soc. 2001, 123, 12798–12801; 11749537 10.1021/ja016812s

[16b] S. Sun , H. Zeng , J. Am. Chem. Soc. 2002, 124, 8204–8205. 12105897 10.1021/ja026501x

[17a] T. J. Daou , G. Pourroy , S. Bégin-Colin , J. M. Grenèche , C. Ulhaq-Bouillet , P. Legaré , P. Bernhardt , C. Leuvrey , G. Rogez , Chem. Mater. 2006, 18, 4399–4404;

[17b] J. Li , X. Shi , M. Shen , Part. Part. Syst. Charact. 2014, 31, 1223–1237.

[18] G. Salazar-Alvarez , M. Muhammed , A. A. Zagorodni , Chem. Eng. Sci. 2006, 61, 4625–4633.

[19] A. Abou-Hassan , O. Sandre , S. Neveu , V. Cabuil , Angew. Chem. Int. Ed. 2009, 48, 2342–2345; 10.1002/anie.200805933 19222077

[20] J. Bemetz , A. Wegemann , K. Saatchi , A. Haase , U. O. Häfeli , R. Niessner , B. Gleich , M. Seidel , Anal. Chem. 2018, 90, 9975–9982. 30044615 10.1021/acs.analchem.8b02374

[21] N. M. Gribanov , E. E. Bibik , O. V. Buzunov , V. N. Naumov , J. Magn. Magn. Mater. 1990, 85, 7–10.

[22] L. Norfolk , A. E. Rawlings , J. P. Bramble , K. Ward , N. Francis , R. Waller , A. Bailey , S. S. Staniland , Nanomaterials 2020, 9. 10.3390/nano9121729 PMC6955933 31817082

[23] L. Frenz , A. El Harrak , M. Pauly , S. Bégin-Colin , A. D. Griffiths , J.-C. Baret , Angew. Chem. Int. Ed. 2008, 47, 6817–6820; 10.1002/anie.200801360 18646028

[24] K. Kumar , A. M. Nightingale , S. H. Krishnadasan , N. Kamaly , M. Wylenzinska-Arridge , K. Zeissler , W. R. Branford , E. Ware , A. J. deMello , J. C. deMello , J. Mater. Chem. 2012, 22, 4704–4708.

[25] C. D. Ahrberg , J. W. Choi , B. G. Chung , Beilstein J. Nanotechnol. 2018, 9, 2413–2420. 30254836 10.3762/bjnano.9.226 PMC6142730

[26] H. Ma , N. Jin , P. Zhang , Y. Zhou , Y. Zhao , X. Zhang , H. Lü , J. Liu , Chem. Eng. Res. Des. 2019, 144, 247–257.

[27] S. F. Chin , K. S. Iyer , C. L. Raston , M. Saunders , Adv. Funct. Mater. 2008, 18, 922–927.

[28] W.-B. Lee , C.-H. Weng , F.-Y. Cheng , C.-S. Yeh , H.-Y. Lei , G.-B. Lee , Biomed. Microdevices 2009, 11, 161–171. 18756355 10.1007/s10544-008-9221-4

[29] M. O. Besenhard , A. P. LaGrow , A. Hodzic , M. Kriechbaum , L. Panariello , G. Bais , K. Loizou , S. Damilos , M. Margarida Cruz , N. T. K. Thanh , A. Gavriilidis , Chem. Eng. J. 2020, 399, 125740.

[30a] A. P. LaGrow , M. O. Besenhard , A. Hodzic , A. Sergides , L. K. Bogart , A. Gavriilidis , N. T. K. Thanh , Nanoscale 2019, 11, 6620–6628; 30896010 10.1039/c9nr00531e

[30b] J. Baumgartner , A. Dey , P. H. H. Bomans , C. Le Coadou , P. Fratzl , N. A. J. M. Sommerdijk , D. Faivre , Nat. Mater. 2013, 12, 310–314. 23377292 10.1038/nmat3558

[31] M. Vallet-Regí , M. Colilla , I. Izquierdo-Barba , M. Manzano , Molecules 2018, 23, 47. 10.3390/molecules23010047 PMC5943960 29295564

[32] G. Martínez-Edo , A. Balmori , I. Pontón , A. Martí del Rio , D. Sánchez-García , Catalysts 2018, 8, 617.

[33] M. H. Nayfeh , L. Mitas , in Nanosilicon (Ed.: V. Kumar ), Elsevier, Amsterdam, 2008, pp. 1–78.

[34] A. Burns , H. Ow , U. Wiesner , Chem. Soc. Rev. 2006, 35, 1028–1042. 17057833 10.1039/b600562b

[35] W. Stöber , A. Fink , E. Bohn , J. Colloid Interface Sci. 1968, 26, 62–69.

[36] S. A. Khan , A. Günther , M. A. Schmidt , K. F. Jensen , Langmuir 2004, 20, 8604–8611. 15379481 10.1021/la0499012

[37] J. B. Wacker , I. Lignos , V. K. Parashar , M. A. M. Gijs , Lab Chip 2012, 12, 3111–3116. 22766615 10.1039/c2lc40300e

[38] L. Gomez , M. Arruebo , V. Sebastian , L. Gutierrez , J. Santamaria , J. Mater. Chem. 2012, 22, 21420–21425.

[39] K. Shiba , T. Sugiyama , T. Takei , G. Yoshikawa , Chem. Commun. 2015, 51, 15854–15857. 10.1039/c5cc07230a 26376831

[40] Y. Nie , N. Hao , J. X. J. Zhang , Sci. Rep. 2017, 7, 12616. 28974729 10.1038/s41598-017-12856-9 PMC5626769

[41] N. Hao , Y. Nie , J. X. J. Zhang , ACS Sustainable Chem. Eng. 2018, 6, 1522–1526.

[42] T. Zhang , Y. Wang , G. Luo , S. Bai , Chem. Eng. J. 2014, 258, 327–333.

[43a] R. Vittal , K.-C. Ho , Renewable Sustainable Energy Rev. 2017, 70, 920–935;

[43b] F. Rahman , Opt. Eng. 2019, 58, 010901;

[43c] X. Chen , Z. Wu , D. Liu , Z. Gao , Nanoscale Res. Lett. 2017, 12, 143; 28235375 10.1186/s11671-017-1904-4 PMC5319938

[43d] L. Zhu , W. Zeng , Sens. Actuators A 2017, 267, 242–261;

[43e] A. Moezzi , A. M. McDonagh , M. B. Cortie , Chem. Eng. J. 2012, 185–186, 1–22.

[44] L. Wang , M. Muhammed , J. Mater. Chem. 1999, 9, 2871–2878.

[45] H. W. Kang , J. Leem , S. Y. Yoon , H. J. Sung , Nanoscale 2014, 6, 2840–2846. 24469327 10.1039/c3nr06141h

[46] A. Baruah , A. Jindal , C. Acharya , B. Prakash , S. Basu , A. K. Ganguli , J. Micromech. Microeng. 2017, 27, 035013.

[47] N. Hao , Z. Xu , Y. Nie , C. Jin , A. B. Closson , M. Zhang , J. X. J. Zhang , Chem. Eng. J. 2019, 378, 122222. 32831625 10.1016/j.cej.2019.122222 PMC7441810

[48] S. N. Khadzhiev , K. M. Kadiev , G. P. Yampolskaya , M. K. Kadieva , Adv. Colloid Interface Sci. 2013, 197–198, 132–145. 10.1016/j.cis.2013.05.003 23768407

[49] Y. Wang , X. Zhang , A. Wang , X. Li , G. Wang , L. Zhao , Chem. Eng. J. 2014, 235, 191–197.

[50] B. G. Zukas , N. R. Gupta , Ind. Eng. Chem. Res. 2017, 56, 7184–7191.

[51] A. Schejn , M. Frégnaux , J.-M. Commenge , L. Balan , L. Falk , R. Schneider , Nanotechnology 2014, 25, 145606. 24633321 10.1088/0957-4484/25/14/145606

[52] D. Dastan , P. U. Londhe , N. B. Chaure , J. Mater. Sci. Mater. Electron. 2014, 25, 3473–3479.

[53] X. Chen , S. S. Mao , Chem. Rev. 2007, 107, 2891–2959. 17590053 10.1021/cr0500535

[54a] B. F. Cottam , S. Krishnadasan , A. J. deMello , J. C. deMello , M. S. P. Shaffer , Lab Chip 2007, 7, 167–169; 17268617 10.1039/b616068a

[54b] Z. Li , L. Yaogang , Z. Qinghong , S. Guoying , W. Hongzhi , Chem. Lett. 2011, 40, 1371–1373;

[54c] A. M. Nightingale , S. H. Krishnadasan , D. Berhanu , X. Niu , C. Drury , R. McIntyre , E. Valsami-Jones , J. C. deMello , Lab Chip 2011, 11, 1221–1227; 21180744 10.1039/c0lc00507j

[54d] M. Baghbanzadeh , T. N. Glasnov , C. O. Kappe , J. Flow Chem. 2013, 3, 109–113;

[54e] J. Beyer , A. Mamakhel , F. Søndergaard-Pedersen , J. Yu , B. B. Iversen , Nanoscale 2020, 12, 2695–2702. 31942897 10.1039/c9nr09069j

[55] H. Wang , H. Nakamura , M. Uehara , M. Miyazaki , H. Maeda , Chem. Commun. 2002, 1462–1463. 10.1039/b203478f 12189844

[56] A. Baruah , A. Singh , V. Sheoran , B. Prakash , A. K. Ganguli , Mater. Res. Express 2018, 5, 075019.

[57a] T. Montini , M. Melchionna , M. Monai , P. Fornasiero , Chem. Rev. 2016, 116, 5987–6041; 27120134 10.1021/acs.chemrev.5b00603

[57b] M. Cargnello , J. J. D. Jaén , J. C. H. Garrido , K. Bakhmutsky , T. Montini , J. J. C. Gámez , R. J. Gorte , P. Fornasiero , Science 2012, 337, 713–717. 22879514 10.1126/science.1222887

[58] A. Dhall , W. Self , Antioxidants 2018, 7, 97. 10.3390/antiox7080097 PMC6116044 30042320

[59] K. Zhou , X. Wang , X. Sun , Q. Peng , Y. Li , J. Catal. 2005, 229, 206–212.

[60] H. Yao , Y. Wang , G. Luo , Ind. Eng. Chem. Res. 2017, 56, 4993–4999.

[61] N.-Y. T. Nguyen , N. Grelling , C. L. Wetteland , R. Rosario , H. Liu , Sci. Rep. 2018, 8, 16260. 30389984 10.1038/s41598-018-34567-5 PMC6214931

[62] K. J. Klabunde , D. G. Park , J. V. Stark , O. Koper , S. Decker , Y. Jiang , I. Lagadic , in Fine Particles Science and Technology: From Micro to Nanoparticles (Ed.: E. Pelizzetti ), Springer Netherlands, Dordrecht, 1996, pp. 691–706.

[63] C. Henrist , J. P. Mathieu , C. Vogels , A. Rulmont , R. Cloots , J. Cryst. Growth. 2003, 249, 321–330.

[64] J. A. Wang , O. Novaro , X. Bokhimi , T. López , R. Gómez , J. Navarrete , M. E. Llanos , E. López-Salinas , J. Phys. Chem. B 1997, 101, 7448–7451.

[65] D. V. R. Kumar , B. L. V. Prasad , A. A. Kulkarni , Ind. Eng. Chem. Res. 2013, 52, 17376–17382.

[66] P. Dhanasekaran , S. R. Williams , D. Kalpana , S. D. Bhat , RSC Adv. 2018, 8, 472–480.

[67] S. A. Khan , S. B. Khan , A. M. Asiri , I. Ahmad , Nanoscale Res. Lett. 2016, 11, 345–345. 27460593 10.1186/s11671-016-1525-3 PMC4961655

[68] A. Pastre , O. Cristini-Robbe , L. Bois , F. Chassagneux , D. Branzea , A. Boé , C. Kinowski , K. Raulin , N. Rolland , R. Bernard , Mater. Res. Express 2016, 3, 015002.

[69] A. H. M. Al-Antaki , X. Luo , X. Duan , R. N. Lamb , W. D. Hutchison , W. Lawrance , C. L. Raston , ACS Omega 2019, 4, 13577–1358. 31460487 10.1021/acsomega.9b01983 PMC6705240

